# Supplementary material for: Factors Associated with 90-Day Mortality in Invasively Ventilated Patients with COVID-19 in Marseille, France
Source: J Clin Med. 2021 Nov 30;10(23):5650. doi: 10.3390/jcm10235650 (PMC8658321; doi:10.3390/jcm10235650)
Supplement: Supplementary file 1 [file jcm-10-05650-s001.zip › jcm-1455069-supplementary.pdf]

	O2 flow (L/min)	Approximated FiO2 (%)
**Nasal cannula**	1	24
	2	28
	3	32
	4	36
	5	40
	6	44
**Mask**	5-6	40
	6-7	50
	7-8	60
**Mask with reservoir**	6	60
	7	70
	8	80
	9	90
	10	95

Figure S1. FiO2 estimation based on O2 flow (L/min) (conversion table from the Extended Prevalence of Infection in Intensive Care (EPIC II) study [1]).

1. The RECOVERY Collaborative Group. Dexamethasone in Hospitalized Patients with Covid-19—Preliminary Report. *N. Engl. J. Med.* **2021**, *384*, 693–704, doi:10.1056/NEJMoa2021436.

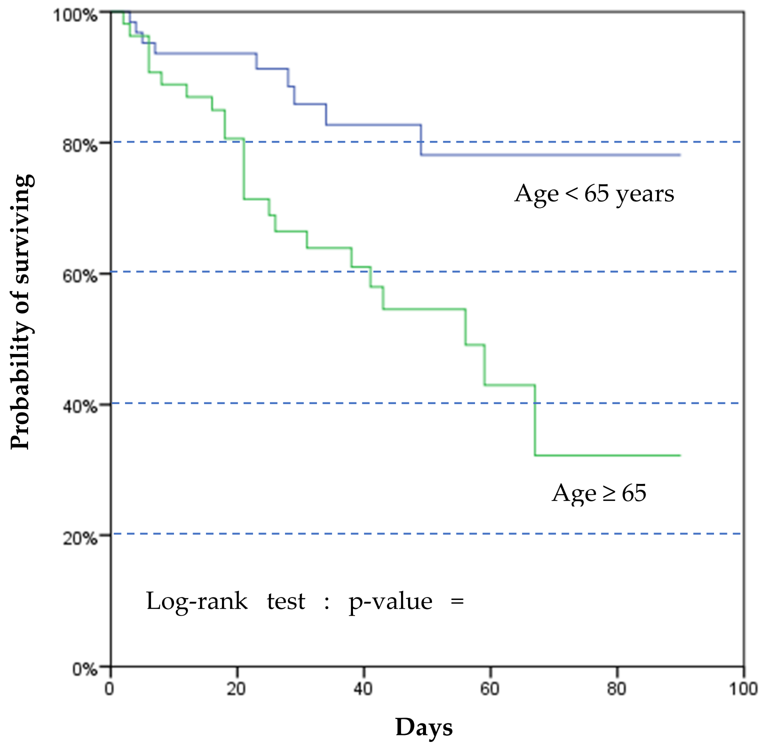

No. at risk

Age < 65 y 63 59 55 54 54
54

Age ≥ 65 y 54 44 36 32 31
31

Figure S2. Kaplan-Meier analysis of survival during the 90 days following ICU admission, according to age categories (<65 or ≥ 65 years).

Table S1. Complete data, including characteristics, management and outcomes, of the 172 patients according to their 90-day survival status.

	Total (N=172)	Survivors (N=135)	Non-survivors (N=37)	p-value
Baseline characteristics				
Age	63(55-71.25)	61(53-68)	72(63-78)	< 0.001
Male	126(73.3)	100(74.1)	26(70.3)	0.643
ABO blood group				
A	54(31.3)	44(32.6)	10(27)	0.471
B	24(20)	20(14.8)	4(10.8)	0.514
O	54(31.3)	40(29.6)	14(37.8)	0.322
AB	2(1.2)	1(0.7)	1(2.7)	0.326
BMI > 25 kg.m²	95(55.2)	80(59.3)	15(40.5)	0.042
Hypertension	92(53.5)	70(51.9)	22(59.5)	0.411
Coronaropathy	14(8.1)	7(5.2)	7(18.9)	0.007
Chronic heart failure	5(2.9)	3(2.2)	2(5.4)	0.307
Diabetes	55(32)	41(30.4)	14(37.8)	0.388
Metabolic syndrome	43(25)	32(23.7)	11(29.7)	0.453
Chronic respiratory disease	29(16.9)	19(14.1)	10(27)	0.062
Chronic kidney disease	13(7.6)	9(6.7)	4(10.8)	0.398
Hepatic cirrhosis	3(1.7)	2(1.5)	1(2.7)	0.615
Neurodegenerative disease	4(2.3)	3(2.2)	1(2.7)	0.864
Active cancer	22(12.8)	15(11.1)	7(18.9)	0.208
Immunodeficiency	35(20.3)	23(17)	12(32.4)	0.04
Smoker (> 20 pack-years)	53(30.8)	42(31.1)	11(29.7)	0.872
Statins intake	32(18.6)	20(14.8)	12(32.4)	0.015
ACE inhibitor	25(14.6)	16(11.9)	9(24.3)	0.059
Angiotensin receptor blockers	32(18.7)	25(18.7)	7(18.9)	0.971
Beta blockers	24(14)	19(14.2)	5(13.5)	0.918
Anticoagulants	7(4.1)	6(4.4)	1(2.7)	0.635
Systemic steroids intake	11(6.4)	6(4.4)	5(13.5)	0.046
Charlson index	3(1-4)	2(1-4)	4(3-5)	< 0.001
SAPS II score	30(24-38)	28(23.25-34)	38(35-44.75)	< 0.001
SOFA score	3(2-6)	3(2-5)	6(3-7)	0.008
NEWS score	6(4-8)	6(4-8)	6(4.25-6.75)	0.853
APACHE II score	10(7-13)	8(7-12)	14(11-15)	0.001
CURB 65	2(1-3)	1(1-2)	3(1.5-3)	0.008
MuLBSTA score	10.5(7-13)	9(7-13)	11(9-13)	0.063
Murray score	3(2.5-3.3)	3(2-3.15)	3(2.7-3.3)	0.086
ROX index	5.5(3.4-9.8)	5.9(3.6-10.2)	4.6(2.7-6.9)	0.045
CT severity visual scoring				
Minor	11(6.4)	8(5.9)	3(8.1)	0.548
Moderate	44(25.6)	38(28.1)	6(16.2)	0.184
Severe	81(69.2)	64(47.4)	17(45.9)	0.808
Total lungs volume on CT (cm³)	3233(2463-4095)	3065(2396-4003)	3743(3261-4158)	0.572
Lesions/lungs ratio on CT (%)				
Ground glass/lungs ratio	21.3(9.9-31.8)	20.8(10.3-33.6)	21.5(7.2-28.2)	0.402
Condensations / lungs ratio	5.2(2.2-10.5)	5.6(2.5-11.8)	2.9(0.4-8.2)	0.05

All lesions / lungs ratio	27.2(13.2-41.8)	27.4(13.7-43.5)	27.2(9.2-35.4)	0.292
Duration between first positive and first negative PCR (days)	11(6-17)	11(5.5-13)	11.5(6.25-20.75)	0.606
Biology (worst value during first 48H after ICU admission)				
Lymphocyte count (x10^9/L)	0.7(0.53-0.99)	0.74(0.56-1.1)	0.59(0.44-0.78)	0.026
Neutrophil to Lymphocyte Ratio	10(6.3-13.8)	8.7(5.7-13.1)	12.5(10-19.6)	0.001
Platelet to Lymphocyte Ratio	333(248-485)	342(228-460)	324(263-514)	0.737
Monocyte to Lymphocyte Ratio	0.63(0.44-0.91)	0.62(0.44-0.91)	0.68(0.49-0.9)	0.77
D-Dimers (mg/L)	2.45(1.29-4.07)	2.18(1.24-4)	4(1.89-5)	0.036
Fibrinogen (g/L)	7.8(6.7-9.2)	7.7(6.7-9)	7.9(6.6-9.3)	0.807
CRP (mg/L)	174(106-275)	150(103-241)	278(152-324)	0.004
Creatinine (µmol/L)	81(64-118)	76(61-107)	99(78-152)	0.002
Albumin (g/L)	33(28.3-36)	32.6(28-36)	33(30-36)	0.513
Troponin	16.5(9-32)	14(8-27.5)	25(15.25-74.25)	0.001
LDH (UI/L)	434(346-530)	415(334-498)	535(412-634)	0.001
Management in ICU				
Limitation on invasive ventilation	7(4.4)	2(1.7)	5(13.5)	0.002
Use of non-invasive ventilation	26(15.2)	21(15.7)	5(13.5)	0.746
Use of high-flow oxygen	110(64.3)	87(64.9)	23(62.2)	0.756
Vasopressors	106(61.6)	74(54.8)	32(86.5)	< 0.001
Inotropic agent	3(1.7)	1(0.7)	2(5.4)	0.055
Renal replacement therapy	20(11.6)	12(8.9)	8(21.6)	0.032
Hydroxychloroquine (10 days) + azithromycin (5 days)	68(39.5)	57(42.2)	11(29.7)	0.169
Remdesivir	0(0)	0(0)	0(0)	-
Lopinavir-ritonavir	20(11.6)	15(11.1)	5(13.5)	0.686
Anakinra	21(12.2)	18(13.3)	3(8.1)	0.390
Tocilizumab	4(2.3)	4(3)	0(0)	0.289
Ruxolitinib	15(8.7)	13(9.6)	2(5.4)	0.42
Complications				
Bacterial coinfection	89(51.7)	66(48.9)	23(62.2)	0.152
Septic shock	47(27.3)	32(23.7)	15(40.5)	0.043
Venous thrombosis or pulmonary embolism	38(22.1)	31(23)	7(18.9)	0.559
Arterial thrombosis	10(5.8)	4(3)	6(16.2)	0.002
Severe bleeding event	25(14.5)	17(12.6)	8(21.6)	0.167
Delirium	22(12.8)	19(14.1)	3(8.1)	0.336
Outcomes				
Length of stay in ICU (days)	18.5(5.75-39)	19(5.5-41.5)	18(6-29)	0.378
Length of in hospital (days)	26(13-43.5)	29(14-47)	21(8-33)	0.09
28-day mortality	26(15.1)	0(0)	26(70)	-

Table S2. Complete data, including characteristics, management and outcomes, of the 117 patients on invasive mechanical ventilation according to their 90-day survival status.

	Total (N=117)	Survivors (N=85)	Non-survivors (N=32)	p-value
Baseline characteristics				
Age	63(56-72)	61(54-67)	71.5(62.75-77)	<0.001
Male	88(75.2)	64(75.3)	24(75.0)	0.974
ABO blood group				
A	42(35.9)	32(37.6)	10(31.2)	0.605
B	17(14.5)	13(15.3)	4(12.5)	0.419
O	41(35)	30(35.3)	11(34.4)	0.366
AB	2(1.7)	1(1.2)	1(3.1)	0.218
BMI > 25 $kg.m^2$	73 (62.4)	59(69.4)	14 (43.8)	0.011
Hypertension	68(58.1)	48(56.5)	20(62.5)	0.556
Coronaropathy	11(9.4)	6(7.1)	5(15.6)	0.157
Chronic heart failure	3(2.6)	2(2.4)	1(3.1)	0.814
Diabetes	24(20.5)	15(17.6)	9(28.1)	0.211
Metabolic syndrome	34(29.1)	29(34.1)	5(15.6)	0.05
Chronic respiratory disease	22(18.8)	15(17.6)	7(21.9)	0.602
Chronic kidney disease	11(9.4)	8(9.4)	3(9.4)	0.995
Hepatic cirrhosis	1(0.9)	1(1.2)	0(0)	0.538
Neurodegenerative disease	1(0.9)	1(1.2)	0(0)	0.538
Active cancer	15(12.8)	10(11.8)	5(15.6)	0.578
Immunodeficiency	24(20.5)	15(17.6)	9(28.1)	0.211
Smoker (> 20 pack-years)	31(26.5%)	21(24.7%)	10(31.3%)	0.475
Statins intake	23(19.7)	12(14.1)	11(34.4)	0.014
ACE inhibitor	22(19)	13(15.5)	9(28.1)	0.12
Angiotensin receptor blockers	20(17.2)	15(17.9)	5(15.6)	0.776
Beta blockers	19(16.4)	14(16.7)	5(15.6)	0.892
Anticoagulants	6(5.1)	5(5.9)	1(3.1)	0.547
Systemic steroids intake	9(7.7)	6(7.1)	3(9.4)	0.675
Charlson index	3(2-4)	2.5(0.8-3.2)	4.2(2.6-5.8)	<0.001
SAPS II score	34(27-40)	30.5(25-38.25)	38(35-45)	0.02
SOFA score	5(3-7)	4(3-7)	5(3-6)	0.966
NEWS score	7(5-8)	7(5-8)	6(4-7)	0.203
APACHE II score	12(8-15)	10(8-13)	14(11-15.25)	0.003
CURB 65	2(1-3)	2(1-2)	3(1.5-3)	0.014
MuLBSTA score	11(8-13)	11(7.25-13)	11(9-13)	0.479
Murray score	3(2.7-3.3)	3(2.7-3.3)	3(2.7-3.3)	0.679
ROX index	4.4(3.1-8.2)	4.4(3.3-8.2)	4.3(2.7-7.5)	0.704
CT severity visual scoring				
Minor	6(5.1)	3(3.5)	3(9.3)	0.182
Moderate	26(22.2)	20(23.5)	6(18.8)	0.613
Severe	54(46.1)	40(47)	14(43.8)	0.824
Total lungs volume on CT (cm^3)	3257(2447-4016)	3142(2285-3804)	3742(3275-4158)	0.01
Lesions/lungs ratio on CT (%)				
Ground glass/lungs ratio	24.8(12.6-34.4)	26.9(13.6-36.6)	21.5(7.1-28.2)	0.862
Condensations / lungs ratio	5.1(2.1-12.4)	6.6(3.1-16)	2.1(0.3-8.2)	0.495

All lesions / lungs ratio	31.8(15.6-46.6)	34.1(19.9-49.7)	26.8(9.2-36.3)	0.034
Days from symptoms onset to intubation	9(6-11.25)	8.5(6-11)	9(6-12)	0.3
Days from ICU admission to intubation	0(0-1)	1(0-1)	0(0-1)	0.364
Duration between first positive and first negative PCR (days)	14(7-18)	14(9-18)	13(7-21.5)	0.892
Biology (worst value during first 48H after ICU admission)				
Lymphocyte count (x10⁹/L)	0.69(0.5-0.95)	0.7(0.53-1)	0.61(0.44-0.78)	0.382
Neutrophil to Lymphocyte Ratio	11.1(8.3-15.5)	10.6(7.5-15.3)	12.6(10.5-19.8)	0.005
Platelet to Lymphocyte Ratio	336(260-506)	347(254-500)	324(268-508)	0.857
Monocyte to Lymphocyte Ratio	0.73(0.5-1)	0.71(0.48-1)	0.76(0.51-0.91)	0.846
D-Dimers (mg/L)	3.44(1.64-5)	3.34(1.52-5)	4(1.84-5)	0.9
Fibrinogen (g/L)	8.1(6.9-9.4)	8.1(7.1-9.5)	8(6.9-9.4)	0.995
CRP (mg/L)	201(126-302)	179(120-248)	283(162-324)	0.29
Creatinine (µmol/L)	85(67-138)	84(65-127)	98(78-153)	0.048
Albumin (g/L)	33(28-36)	33(27-36)	34(31-36)	0.148
Troponin	21(11.75-36)	18(10-32)	25(14.5-59)	0.045
LDH (UI/L)	447(368-535)	435(347-491)	539(443-635)	0.005
Ferritin (ng/mL)	1418(968-2321)	1175(950-1778)	2728(2342-6049)	0.021
Invasive ventilation parameters during first 24H				
PaO2/FiO2	130 (100-180)	140(100-180)	120(100-160)	0.457
Tidal volume (mL)	420(385-440)	410(380-430)	420(397.5-450)	0.149
PEEP (cmH2O)	12(10.3-14)	12(12-14)	12(10-15)	0.483
Plateau pressure (cmH2O)	25(21.7-28.3)	23.5 (21.0-28.2)	26.0 (23.6-28.8)	0.353
Respiratory compliance (mL/cmH2O)	33(28.2-45)	40(29-46)	31(27-40)	0.018
Mechanical power (J/min)	15.6(13.3-19.6)	15.5(13.5-19.1)	15.9(12-19.8)	0.836
Management in ICU				
Use of non-invasive ventilation before intubation	16(13.7)	12(14.1)	4(12.5)	0.693
Use of high-flow oxygen before intubation	69(59)	51(60)	18(56.2)	0.589
Neuromuscular blockade	113(96.6)	82(96.5)	31(96.9)	0.923
Prone positioning	99(84.6)	72(84.7)	27(84.4)	0.965
Nitric oxide	42(35.9)	28(32.9)	14(48.3)	0.277
Tracheotomy	50(42.7)	45(52.9)	5(15.6)	< 0.001
ECMO	23(19.7)	18(21.2)	5(15.6)	0.501
Vasopressors	105(89.7)	74(87.1)	31(96.9)	0.122
Inotropic agent	3(2.6)	1(1.2)	2(6.3)	0.122
Renal replacement therapy	20(17.1)	12(14.1)	8(25)	0.167
Hydroxychloroquine (10 days) + azithromycin (5 days)	45(38.5%)	36(42.4)	9(28.1)	0.159
Remdesivir	0(0)	0(0)	0(0)	-
Lopinavir-ritonavir	18(15.4)	13(15.3)	5(15.6)	0.965
Anakinra	16(13.7)	13(15.3)	3(9.4)	0.406
Tocilizumab	3(2.6)	3(3.5)	0(0)	0.282
Ruxolitinib	13(11.1)	11(12.9)	2(6.9)	0.305
Complications				
Ventilator associated pneumonia	71(60.7)	53(62.4)	18(56.2)	0.547
Bacterial coinfection	86(73.5)	64(75.3)	22(68.8)	0.475
Septic shock	47(40.2)	32(37.6)	15(46.9)	0.364

Venous thrombosis or pulmonary embolism	35(29.9)	29(34.1)	6(18.8)	0.106
Arterial thrombosis	10(8.5)	4(4.7)	6(18.8)	0.015
Severe bleeding event	25(21.4)	17(20)	8(25)	0.556
Delirium	21(17.9)	19(22.4)	2(6.3)	0.043
Outcomes				
Duration of ventilation (days)	20(9-33)	21(11-34)	18(6.75-25.25)	0.06
Ventilator-free days at d28 (days)	2(1-7)	4(1-7)	0(0-1)	<0.001
Length of stay in ICU (days)	29(17-46)	33(19-53)	21(6.75-31.75)	0.02
Length of in hospital (days)	37(24-53)	42(29-57)	25(8.75-38.25)	<0.001
28-day mortality	21(17.9)	0(0)	21(60)	-
